# Basis of the Massachusetts Reference Dose and Drinking Water Standard for Perchlorate

**DOI:** 10.1289/ehp.0900635

**Published:** 2009-07-13

**Authors:** Tsedash Zewdie, C. Mark Smith, Michael Hutcheson, Carol Rowan West

**Affiliations:** Massachusetts Department of Environmental Protection, Office of Research and Standards, Boston, Massachusetts, USA

**Keywords:** drinking water, neurodevelopment, perchlorate, reference dose, thyroid

## Abstract

**Objective:**

Perchlorate inhibits the uptake of iodide in the thyroid. Iodide is required to synthesize hormones critical to fetal and neonatal development. Many water supplies and foods are contaminated with perchlorate. Exposure standards are needed but controversial. Here we summarize the basis of the Massachusetts (MA) perchlorate reference dose (RfD) and drinking water standard (DWS), which are considerably lower and more health protective than related values derived by several other agencies. We also review information regarding perchlorate risk assessment and policy.

**Data sources:**

MA Department of Environmental Protection (DEP) scientists, with input from a science advisory committee, assessed a wide range of perchlorate risk and exposure information. Health outcomes associated with iodine insufficiency were considered, as were data on perchlorate in drinking water disinfectants.

**Data synthesis:**

We used a weight-of-the-evidence approach to evaluate perchlorate risks, paying particular attention to sensitive life stages. A health protective RfD (0.07 μg/kg/day) was derived using an uncertainty factor approach with perchlorate-induced iodide uptake inhibition as the point of departure. The MA DWS (2 μg/L) was based on risk management decisions weighing information on perchlorate health risks and its presence in certain disinfectant solutions used to treat drinking water for pathogens.

**Conclusions:**

Current data indicate that perchlorate exposures attributable to drinking water in individuals at sensitive life stages should be minimized and support the MA DEP perchlorate RfD and DWS. Widespread exposure to perchlorate and other thyroid toxicants in drinking water and foods suggests that more comprehensive policies to reduce overall exposures and enhance iodine nutrition are needed.

Perchloric acid and most perchlorate salts, which are widely used as oxidants in aerospace fuels and explosives, readily dissolve in water generating the perchlorate anion, ClO_4_^−^ (perchlorate). Perchlorate is persistent, exhibits poor affinity for soils, is highly mobile in groundwater, and has been detected nationally in groundwater and surface water associated with the aerospace industry, military bases, and blasting sites [Massachusetts Department of Environmental Protection (MA DEP) 2005; U.S. Environmental Protection Agency (EPA) 2002c]. Other potential sources include fireworks, hypochlorite water disinfection products, and certain manufacturing processes (MA DEP 2005; U.S. EPA 2002c). Perchlorate compounds also occur naturally in Chilean nitrate fertilizers, evaporite soils, and the atmosphere (Dasgupta et al. 2005).

Perchlorate is of toxicological concern because it interferes with iodide uptake in the thyroid and can disrupt the synthesis of thyroid hormones critical to normal growth, development, and other physiological functions [Greer et al. 2002; National Research Council (NRC) 2005; Yu et al. 2002]. Because of widespread environmental contamination, people can be exposed to perchlorate found in drinking water, breast milk, foods, and beverages. Many food items accumulate perchlorate from contaminated soil and water (Yu et al. 2004), and human biomonitoring data have demonstrated widespread exposures attributed to dietary sources [Blount et al. 2006b; El Aribi et al. 2006; Food and Drug Administration (FDA) 2009; Valentin-Blasini et al. 2005].

In 2001 and 2002, perchlorate was detected in groundwater at the Massachusetts Military Reservation and the town of Bourne on Cape Cod, respectively. Because no state or federal drinking water standard (DWS) (or maximum contaminant level) was available, MA DEP, with input from an independent science advisory committee (SAC), derived a reference dose (RfD) of 0.07 μg/kg/day for perchlorate using a weight-of-the-evidence approach that considered data from mode of action, human, and animal studies. An RfD provides an estimate of the daily dose of a chemical likely to be without deleterious effects over a lifetime, with uncertainty of about an order of magnitude (U.S. EPA 2002a). Following extensive public input, a MA perchlorate DWS of 2 μg/L was adopted in 2006. In this review, we summarize the scientific basis and risk management decisions supporting these values and compare the MA values with those derived by the NRC (RfD = 0.7 μg/kg/day), U.S. EPA [interim drinking water health advisory (IDWHA) = 15 μg/L], and California (DWS = 6 μg/L).

## Key Studies, Data Sources, and Toxicological Considerations for Perchlorate Risk Assessment

### Toxicokinetics

Ingested perchlorate is extensively absorbed from the gastrointestinal system and accumulates in the thyroid (MA DEP 2006; NRC 2005; U.S. EPA 2002c). Most absorbed perchlorate is excreted unchanged in the urine, with a biological half-life of about 8 hr (Lamm et al. 1999; Wolff 1998). Biphasic clearance with a second-phase half-life of 70–80 hr in rats has been reported (Von Burg 1995). This pattern is consistent with a multicompartment model with accumulation and slow clearance from at least one compartment, likely to include the thyroid.

Perchlorate crosses the placenta and has been detected in amniotic fluid (Blount and Valenti-Blasini 2006). It is also secreted into human breast milk and may reduce iodine levels in the milk (Dasgupta et al. 2008). All breast milk samples collected from 36 women from 18 states contained perchlorate at concentrations ranging from 1.4 to 92.2 μg/L (mean = 10.5 μg/L; median = 3.25 μg/L) (Kirk et al. 2005). Perchlorate in 147 samples of breast milk from 10 women ranged from 0.5 to 39.5 μg/L (mean ± SD = 5.8 ± 6.2 μg/L; median = 4 μg/L) (Kirk et al. 2007). Forty-nine samples of breast milk from women in Boston all contained perchlorate ranging from 1.3 to 411 μg/L (mean ± SD = 33 ± 77 μg/L; median = 9.1 μg/L). No association was found between milk perchlorate and iodine content, but an estimated 47% of the women may have been expressing milk with insufficient iodine (Pearce et al. 2007b). In > 400 samples of breast milk from 13 women, perchlorate ranged from 0.01 to 48 μg/L (mean ± SD = 9.3 ± 7.5 μg/L; median = 7.3 μg/L); compared with iodide, perchlorate was estimated to be preferentially secreted into milk, and breast milk from 12 of the 13 women did not contain adequate iodine (Dasgupta et al. 2008). Preferential secretion of perchlorate over iodide into breast milk would reduce the nursing infant’s iodide intake and exacerbate neonatal thyroid sensitivity to perchlorate in the milk.

### Mode of action

The thyroid gland transports and concentrates iodide from the blood, which is necessary for the synthesis of the thyroid hormones thyroxin (T_4_) and triiodothyronine (T_3_) (NRC 2005). Iodide transport is mediated by the sodium (Na)–iodide (I) symporter (NIS), an integral membrane protein present in the thyroid, the mammary gland, and other tissues (Vieja et al. 2000). Perchlorate competitively inhibits iodide transport by the NIS (Greer et al. 2002; NRC 2005; Wolff 1998) and promotes the discharge of endogenous thyroidal iodide through a mechanism that is not well understood (Bürgi et al. 1974; Wolff 1998; Wyngaarden et al. 1952). These actions reduce iodide availability for synthesis of T_4_ and T_3_ (Greer et al. 2002; Wyngaarden et al. 1952; Yu et al. 2002). Perchlorate is also actively transported into thyrocytes (Dohán et al. 2007; Tran et al. 2008).

Although NIS inhibition is viewed as perchlorate’s primary mode of action, other possible mechanisms that could contribute to its toxicity may exist. Based on a biologically based dose–response model of the hypothalamus– pituitary–thyroid axis and physiologically based pharmacokinetically (PBPK) modeled perchlorate distribution and inhibition of thyroid iodide uptake, a recent assessment concluded that iodide uptake inhibition (IUI) was insufficient to explain observed changes in rat thyroid hormone levels attributable to perchlorate (McLanahan et al. 2009). These results suggest that an additional mechanism of action may exist, perhaps attributable to perchlorate uptake into the thyroid and interference with other targets involved in hormone synthesis and release. The pendrin protein, which mediates iodide transport across the thyroidal apical membrane into the follicular lumen, where it is used in the iodination of thyroglobulin, is one such possible target (MA DEP 2006; Scinicariello et al. 2005). Mutations in the pendrin protein result in defects in iodide transport into the follicular lumen and impaired iodide organification. The resulting iodide accumulation in the thyrocyte may exacerbate perchlorate-induced iodide discharge (Scinicariello et al. 2005). Perchlorate interference with the pendrin protein could similarly exacerbate iodide discharge or directly lead to decrements in organification.

Decreases in the serum levels of thyroid hormones trigger a feedback system involving the hypothalamus–pituitary–thyroid axis (NRC 2005). The hypothalamus uses thyrotropin-releasing hormone (TRH) to control the pituitary gland, which in turn controls the thyroid through thyroid-stimulating hormone (TSH). Secretion of TRH, and hence TSH, is typically increased by low blood levels of thyroid hormones in a classical negative feedback loop. This homeostatic mechanism, as well as the storage of excess thyroid hormone by the healthy mature thyroid, may mitigate the effects of thyroid toxicants (NRC 2005). However, because of individual and life-stage variability in the effectiveness of these homeostatic controls and in thyroid reserve capacities, potential sensitive subgroups must be considered in the derivation of exposure limits for perchlorate (Ginsberg et al. 2007; MA DEP 2006; Savin et al. 2003; Scinicariello et al. 2005; van den Hove et al. 1999; Zoeller 2003).

### Sensitive subgroups

Based on many lines of evidence, fetuses, premature infants, and newborns are at greater risk of perchlorate toxicity. Appropriate levels of thyroid hormones are critical to the development of various organs, especially the brain, which occurs during fetal growth and childhood (Howdeshell 2002; Zoeller et al. 2002). During the fetal and neonatal periods, the thyroid gland is incompletely developed, increasing susceptibility to toxicants that disrupt thyroid function. These life stages exhibit low thyroid hormone storage capacity, estimated at < 1 day in neonates (Savin et al. 2003; van den Hove et al. 1999), as well as thyroid hormone turnover rates two to three times faster than in adults (van den Hove et al. 1999) and poorly developed adaptive mechanisms (Pedraza et al. 2006). Although the impact of thyroid toxicants on the fetus can be buffered by the thyroid hormone synthesis and reserve capacities of the mother, iodide insufficiency and pregnancy-related stresses on maternal thyroid function increase the potential for perchlorate effects during fetal development (Ginsberg et al. 2007; Glinoer 2001). In the case of the nursing infant, perchlorate secretion into breast milk and reported interference with iodide transport into the milk (Dasgupta et al. 2008) present a potential double risk attributable to maternal perchlorate exposure.

Iodine-deficient populations are also likely to be at greater risk of perchlorate toxicity and provide information relevant to understanding potential perchlorate effects. Iodine deficiency is strongly associated with poor neurodevelopmental outcomes (Bleichrodt and Born 1994). In areas where iodine intake is marginal (< 100 μg/day), low serum T_4_ and T_3_ levels (hypothyroidism), enlarged thyroid, and goiter were often detected in pregnant women, with impaired intellectual and physical development in offspring (Caron et al. 1997; Glinoer 2001). Children born to iodine-deficient mothers had intelligence quotients (IQs) 5–13 points lower than children born to iodine-sufficient mothers (Bleichrodt and Born 1994; Van den Briel et al. 2000). Moderate iodine deficiency was associated with a 4-fold increase in risk of low IQ (Pineda-Lucatero et al. 2008), and in an area of mild to moderate iodine deficiency, children with urinary iodine < 100 μg/L had significantly lower IQs than did those with concentrations > 100 μg/L (Santiago-Fernandez et al. 2004). In iodine-sufficient areas, children of women with free T_4_ levels below the 5th and 10th percentile at 12 weeks of gestation had significantly lower scores on psychomotor development tests at 10 months of age compared with children of mothers with higher T_4_ (Pop et al. 1999). Severe iodine deficiency (< 20 μg/day iodine intake) has been associated with stillbirths, congenital anomalies, perinatal mortality, and cretinism (Delange 2000). Taken together, these studies indicate that functional iodine deficiency, as could be caused or exacerbated by perchlorate exposures *in utero* and during early development, may lead to adverse neurodevelopmental outcomes.

### Clinical studies

Six controlled clinical studies have investigated thyroid effects following oral exposures to perchlorate [see Supplemental Material, Section B and Table S1, available online (doi:10.1289/ehp.0900635.S1 via http://dx.doi.org)]. All of these studies included small study populations ranging from 5 to 37 subjects, limiting their overall statistical power to detect effects; none addressed pregnant women, iodine-deficient groups, or children; and only one (Greer et al. 2002) included more than two dose groups.

The Greer et al. (2002) study provides the best available dose–response information on perchlorate-induced IUI and served as the key study in the MA DEP, NRC, and California Environmental Protection Agency (CalEPA) assessments. In this study, perchlorate was administered in drinking water at 0.007, 0.02, 0.1, or 0.5 mg/kg/day to 37 iodine-sufficient healthy male and female volunteers for 14 days, and radioactive iodide uptake (RAIU) was measured at different time points (Greer et al. 2002). Statistically significant IUI was observed in the three highest dose groups. IUI was also observed in the 0.007 mg/kg/day group but was not statistically significant. The low-dose group consisted of only seven subjects, and individual relative uptake values exhibited considerable variability. The authors reported a no observed effect level of 0.007 mg/kg/day (7 μg/kg/day).

### Population studies

Several population studies have investigated perchlorate exposure and thyroid effects. Adverse health effects were not observed in three occupational studies (Braverman et al. 2005; Gibbs et al. 1998; Lamm et al. 1999), although decrements in RAIU and alterations in thyroid hormone levels, which the authors did not consider to be adverse, were reported by Braverman et al. (2005). The applicability of these studies to the broader population is limited because they did not address sensitive life stages or subgroups. They were also cross-sectional in design and subject to survivor bias.

A number of epidemiological studies of ecological design, with exposures largely categorized by perchlorate concentrations in water supplies rather than measurements of individual exposure, have also been completed. Most of these were negative (Crump et al. 2000; Kelsh et al. 2003; Lamm and Doemland 1999; Li et al. 2000a, 2000b, 2001; Morgan and Cassady 2002), with two reporting positive associations between putative perchlorate exposures and thyroid effects (Brechner et al. 2000; Schwartz 2001). Because perchlorate is present in many foods, exposure misclassification in these studies is likely significant, reducing their statistical power and biasing results toward the null hypothesis of no effect.

Several population studies have used urinary perchlorate as a measure of intake, which minimizes exposure misclassification and allows for more robust data analyses (Amitai et al. 2007; Blount et al. 2006a; Gibbs and van Landingham et al. 2008; Steinmaus et al. 2007; Tellez et al. 2005). In an analysis of data from a nationally representative group of 2,820 individuals sampled during the 2001–2002 National Health and Nutrition Examination Survey (NHANES), an association between perchlorate exposure levels experienced in the general U.S. population and decreased serum T_4_ and increased TSH was observed in women with urinary iodine concentrations < 100 μg/L (Blount et al. 2006a). An independent analysis of the 2001–2002 NHANES data reported similar results (Steinmaus et al. 2007). Based on 2001–2002 NHANES data, median U.S. perchlorate intake was estimated to be 0.066 μg/kg/day for adults > 20 years of age (Blount et al. 2007), and about 36% of U.S. women were found to have urinary iodine levels < 100 μg/L (Caldwell et al. 2005).

Other studies have reported no association between urinary perchlorate and thyroid function. Tellez et al. (2005) performed a longitudinal epidemiological study of pregnant women in Chile exposed to perchlorate in drinking water and the diet. The authors concluded that perchlorate in drinking water as high as 114 μg/L did not affect maternal and neonatal thyroid function. However, the applicability of this finding to the United States is uncertain because urinary iodine levels in the study populations were 30–40% higher in each quintile than those reported in NHANES III for pregnant U.S. women (Tellez et al. 2005). In a recent analysis, no associations between free T_4_ or TSH and perchlorate exposure were found in 16 individuals from the Tellez et al. (2005) cohort with urinary iodide < 100 μg/L (Gibbs and van Landingham 2008). Although these results are not supportive of the 2001–2002 NHANES findings, the sample size is very small. A larger study of three European cohorts with median urinary iodine < 100 μg/L found no associations between urinary perchlorate and first-trimester serum thyroid function tests (Pearce et al. 2007a). The significance of these results is difficult to assess because little detail on experimental protocols, study population characteristics, or statistical analyses was provided. In another study, neonatal T_4_ levels were compared between newborns in Israel from areas with drinking water perchlorate concentrations ranging from < 3 to 340 μg/L (Amitai et al. 2007). Maternal exposures were estimated from serum perchlorate concentrations of donors in each area and were consistent with a > 10-fold range in exposures. No association between T_4_ and elevated gestational exposure to perchlorate was observed. The authors noted that the study population was iodine sufficient and stated that their results indicate that the NRC “perchlorate RfD of 0.7 μg/kg/day is likely to be protective of thyroid function in neonates of mothers with adequate iodide intake.” However, they also noted that because of differences in iodine intake and life stages between the studies, their results did not contradict those of Blount et al. (2006a).

In summary, although the epidemiological studies do not paint a consistent picture regarding perchlorate exposure and thyroid effects, the largest and most complete assessments to date, Blount et al. (2006a) and Steinmaus et al. (2007), did find such an association at doses commonly encountered in the general U.S. population.

### Animal studies

In summary, the animal studies have demonstrated IUI, decreased serum T_4_ and T_3_, increased TSH, thyroid hypertrophy and hyperplasia across life stages, and possible alterations in brain morphometry and behavior in rat pups exposed to perchlorate *in utero* and after birth (U.S. EPA 2002c) [see Supplemental Material, Section C (doi:10.1289/ehp.0900635.S1)]. These observations are consistent with perchlorate’s mode of action. In the most sensitive species, changes in thyroid hormones were observed at the lowest dose tested, 0.0085 mg/kg/day (U.S. EPA 2002c).

## MA DEP RfD Derivation

### Key study and effect

We chose Greer et al. (2002) as the key study for deriving the MA perchlorate RfD because it provided the best available human dose–response data. The NRC and CalEPA also used this study (CalEPA 2004; NRC 2005). IUI was selected as the critical effect, which is defined as the first adverse effect, or its known precursor, that occurs in the most sensitive species as the dose rate of the agent increases (U.S. EPA 2002a). IUI is a biochemical change that precedes effects on thyroid hormone status. RfDs have been based on similar precursor biochemical changes, such as plasma or red cell cholinesterase inhibition for various organophosphates (U.S. EPA 2005a).

### Point of departure

MA DEP considered two approaches to derive an RfD for perchlorate: the benchmark dose (BMD) methodology, which uses dose–response modeling at a selected response rate to derive a point of departure (POD) for deriving an RfD and a lowest observed adverse effect level (LOAEL) approach. The U.S. EPA and CalEPA performed BMD analyses on the Greer et al. (2002) data using a 5% IUI response rate. Excluding two statistical outliers, the U.S. EPA calculated a 95% lower one-sided confidence limit on the BMD (BMDL) of 2 μg/kg/day, whereas the CalEPA derived a BMDL of 3.7 μg/kg/day (Ting et al. 2006; U.S. EPA 2002c, 2003). U.S. EPA ultimately adopted the NRC RfD, which relied on the lowest dose in the Greer et al. (2002) study, 0.007 mg/kg/day (7 μg/kg/day), as the POD and treated this value as a no-effect level. CalEPA also treated their BMDL as a no-effect level and relied on this value as the POD in their RfD derivation.

**Figure 1:**
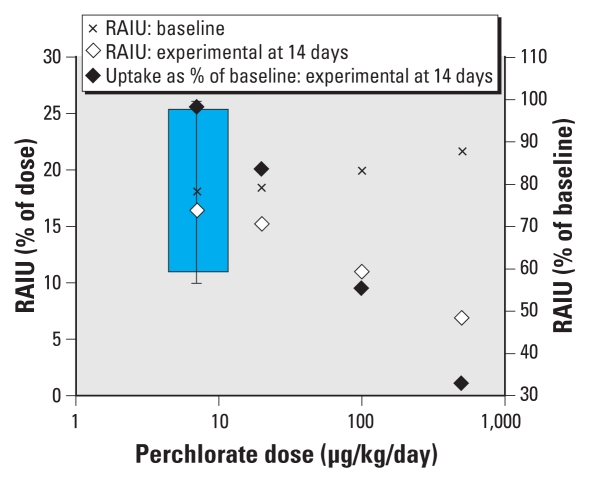
Iodide uptake data from Greer et al. (2002): means with low-dose-group baseline SD. The shaded box to the left demarcates the range of RAIU values (10.9–25.3%) within which differences from baseline for 7 μg/kg/day group cannot be statistically discriminated at an α of 0.05 given sample size of 7 and an SD of 8.2 about the baseline mean.

Because we could identify no objective data delineating a level of IUI that would not elicit downstream effects, we established a POD using the LOAEL approach, which does not necessitate the explicit choice of an IUI level of concern. MA DEP used as the POD the lowest dose in the Greer et al. (2002) study, where nonstatistically significant IUI was observed, and treated this dose as a minimal LOAEL. A minimal designation was used because the effect is not frankly adverse. The SAC concurred with this determination, which was primarily based on three observations. First, the Greer et al. (2002) study had very low statistical power. Using standard sample size calculation approaches (Green 1979; Sokal and Rohlf 1995), the minimum difference in RAIU in the Greer et al. study’s low perchlorate dose group that could be discriminated from the baseline mean ± SD of 18.1 ± 8.2% was ± 40% at an α of 5% (Figure 1). Thus, the Greer et al. (2002) study was unable to reliably detect up to a 40% change in RAIU at the lowest dose tested. Second, in the lowest dose group, several subjects exhibited reduced RAIU (Greer et al. 2002). Third, perchlorate exposures with predicted IUI of < 5% based on PBPK modeling were associated with thyroid hormone alterations in animal bioassays (U.S. EPA 2002c). More recent human data are also consistent with perchlorate effects on the thyroid in sensitive subgroups at doses predicted to cause low levels of IUI. At the median U.S. perchlorate dose of 0.066 μg/kg/day, estimated from the 2001–2002 NHANES data (Blount et al. 2007), IUI of < 1% would be predicted based on recent U.S. EPA modeling (U.S. EPA 2008a, Table 5-4).

### Accounting for uncertainties

Consistent with U.S. EPA and MA DEP protocols, uncertainty factors (UFs) are used to adjust the POD to account for gaps in scientific data and knowledge. MA DEP concluded that a total UF of 100 was appropriate for deriving an RfD for perchlorate. This UF accounts for database insufficiencies as well as extrapolation from results in a small group of healthy adults to sensitive subgroups and from a minimal LOAEL to a no observed adverse effect level (NOAEL). An adjustment for database deficiencies was included due to a lack of data from chronic exposure studies; uncertainty regarding perchlorate interference with iodide transport into breast milk, which could increase the sensitivity of the neonate; and uncertainties relating to perchlorate’s mode of action, immunotoxicity, and possible carcinogenicity.

### RfD calculation

Using the minimum LOAEL of 7 μg/kg/day and a UF of 100 the final MA RfD was calculated as follows:


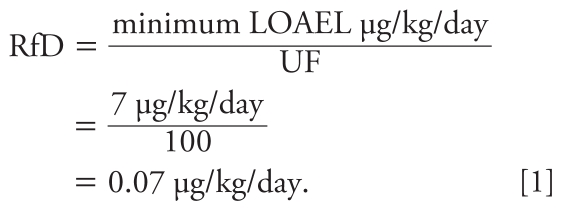


Results from animal studies support this, or an even lower, RfD (U.S. EPA 2002c) [see Supplemental Material, Section C (doi:10.1289/ehp.0900635.S1)]. Most SAC members supported this RfD. One member supported a higher UF of 300, which would result in an RfD of 0.02 μg/kg/day. A second member supported a UF of 30, which would result in an RfD of 0.23 μg/kg/day.

## Health-Based Drinking Water Guidelines

### Approach

The general equation used to convert RfDs to a drinking water guideline (DWG) is


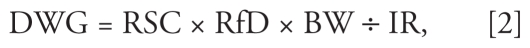


where RfD is the reference dose (micrograms per kilogram per day), RSC is the relative source contribution factor or the fraction of the RfD allocated to drinking water (unitless), BW is body weight (kilograms), and IR is water ingestion rate (liters per day).

The RSC is applied to account for non-drinking water exposures that contribute to overall exposure. When non-drinking water exposures are likely but the data are insufficient to quantify their relative contributions, U.S. EPA recommends a default RSC of 20% (U.S. EPA 1998). When MA DEP derived its DWG for perchlorate in 2005, the available data were insufficient to calculate an RSC, and we opted to use the 20% default. The current database on U.S. perchlorate exposures is more robust. Perchlorate was detected in all 2001–2002 NHANES urine samples, with estimated median and 95th percentile intakes of 0.066 μg/kg/day and 0.234 μg/kg/day, respectively, for adults > 20 years of age (Blount et al. 2007). The 2005–2006 FDA Total Diet Study (TDS) estimated lower bound (average values with nondetects treated as zero) perchlorate exposures attributable to food ranging from 0.08 μg/kg/day for 25- to 30-year-old men to 0.35 μg/kg/day for 2-year-olds (Murray et al. 2008; U.S. EPA 2008a). Using the 2001–2002 NHANES data and restricting the analysis to individuals unlikely to have drinking water exposure, the U.S. EPA estimated mean perchlorate food exposures for pregnant women and the total population to be 0.123 and 0.090 μg/kg/day, and 90th percentile values to be 0.263 and 0.167 μg/kg/day, respectively (U.S. EPA 2008a). Thus, most current estimates of mean and median perchlorate exposures attributable to food exceed the MA RfD of 0.07 μg/kg/day and would support an RSC of < 20%.

### Adult DWG value

Using Equation 2 and a 20% RSC, the health-based DWG for perchlorate equals 0.49 μg/L for a 70-kg adult consuming 2 L water/day. Use of the alternative RfDs supported by some members of the SAC yield DWG values of 0.16 and 1.6 μg/L. Because many of the recent estimates of perchlorate exposures attributable to food exceed the MA RfD, a lower DWG could be supported.

### Neonatal exposure adjustments

Perchlorate risks to neonates are of particular concern because of their limited reserve capacity of thyroid hormones and incompletely developed thyroid functions (Pedraza et al. 2006; Savin et al. 2003; van den Hove et al. 1999). Neonatal exposures also differ from those of adults because of higher liquid intakes per unit of BW and the consumption of breast milk. To address neonatal water ingestion, MA DEP derived a DWG using an infant fluid consumption rate of 0.64 L/day and BW of 4 kg (U.S. EPA 2002b), which results in a higher exposure rate per unit of BW compared with adults. For formula-fed infants, whose exposures attributable to other foods are limited, the perchlorate DWG calculated using these infant parameters, without an RSC, equals 0.44 μg/L.

The situation for the breast-fed infant is more complex, because it requires a DWG for the lactating mother that appropriately accounts for perchlorate entry into breast milk. At the time that MA DEP derived its DWG for perchlorate, we concluded that it was not possible to derive meaningful quantitative estimates of infant perchlorate exposures from breast milk directly attributable to maternal drinking water exposures because of limited data and uncertainties in the PBPK models for fetal and neonatal life stages. More recent data indicate that neonatal exposures to perchlorate in breast milk are significant. The median perchlorate exposure to 2-week-old nursing infants in the United States was predicted to be 0.206 μg/kg/day (95th percentile = 0.744 μg/kg/day) (Ginsberg et al. 2007). Other data indicate that nursing infant perchlorate exposures in the United States can be even higher. Based on a breast milk intake rate of 0.172 L/kg/day and reported median breast milk perchlorate concentrations (Kirk et al. 2005, 2007; Pearce et al. 2007b), we estimate that nursing infant perchlorate intakes in three U.S. cohorts ranged from 0.56 to 1.57 μg/kg/day. Dasgupta et al. (2008) estimated breast milk perchlorate intake rates ranging from 0.3 to 2.1 μg/kg/day, with intakes of 9 of 13 infants exceeding the NRC RfD (NRC 2005). These data support precautionary maternal exposure limits.

## MA Drinking Water Standard

The final MA DWS for perchlorate was established at 2.0 μg/L. This value was the result of a risk management decision that took into account the health-based guidelines derived above and concerns relating to the presence of perchlorate in hypochlorite drinking water disinfection solutions. Although other drinking water treatments exist (e.g., ultraviolet light) where perchlorate would not be a concern, hypochlorite solutions are used at many public drinking water supplies (PDWS). Depending on a number of factors, hypochlorite solutions can undergo oxidative decomposition generating chlorate, ClO_3_^−^ (Gordon and Bubnis 1996). Chlorate can be further oxidized, perhaps catalyzed by trace transition metal contaminants, to generate perchlorate, ClO_4_^−^ (Schumacher 1960). Commercial sodium hypochlorite solutions from MA drinking water and wastewater treatment facilities were found to have perchlorate at levels ranging from < 1 μg/L in newly delivered solutions to 6,750 μg/L in solutions stored for 26 days postdelivery (mean = 2,461 μg/L in the 26-day-old solutions) (Table 1). Similar levels were also detected in household bleach, with higher levels also found in older solutions. Ninety-one percent of 82 commercial sodium hypochlorite drinking water treatment solutions were found to contain perchlorate at concentrations that would result in, at the certified maximum use level of 10 mg/L chlorine, treated drinking water perchlorate concentrations ranging from 0.03 to 29 μg/L (Greiner et al. 2008). At the certified maximum use level, 40% of the hypochlorite solutions tested would lead to finished drinking water perchlorate concentrations in excess of 1 μg/L, and 29% in excess of 2 μg/L (Greiner et al. 2008). Perchlorate from the use of hypochlorite disinfection solutions would add to any existing level of this agent in the source water and could create compliance issues at PDWS attributable to prechlorination, chlorination, and shock chlorination treatments of water supplies to address pathogens.

In light of these findings and to avoid the creation of potential compliance issues that could create disincentives for necessary water supply disinfection, MA DEP established the DWS at 2 μg/L following extensive public hearings. MA DEP concluded that this value would reasonably minimize potential perchlorate exceedences attributable to chlorination, balancing perchlorate exposure and infectious disease control concerns. This value was also determined to be within the range of scientific uncertainty regarding perchlorate toxicity.

## Discussion

The MA RfD and DWS are lower than related values derived by U.S. EPA, NRC, and CalEPA (Table 2). Although all three groups relied on the IUI results of Greer et al. (2002), they differed in their treatment of this end point and in the selection of UFs. Views on the significance of IUI vary. The NRC considered IUI to be a nonadverse precursor effect but relied on this end point as the key effect in their RfD derivation, treating the lowest dose from Greer et al. (2002) as a no-effect level. They further suggested that IUI would be unlikely to lead to adverse effects unless the degree of inhibition was very large (75%) (NRC 2005). CalEPA used a 5% IUI response rate in their BMD modeling (Ting et al. 2006). The U.S. EPA recently concluded that PBPK model-predicted IUI of up to 2.2% in bottle-fed infants would not be significant (U.S. EPA 2008a). However, the level of IUI necessary to cause deleterious downstream effects is unknown, and the available data suggest that small increments in IUI may be significant. In rodents, perchlorate doses estimated to cause PBPK modeled IUI of < 5% were associated with altered thyroid hormone status and possible developmental effects (U.S. EPA 2002c). Additionally, perchlorate intakes estimated from the 2001–2002 NHANES data (Blount et al. 2007) would be predicted to cause IUI of < 1% on the basis of the U.S. EPA modeling (U.S. EPA 2008a). Thyroid hormone alterations in women with urinary iodine < 100 μg/L were associated with perchlorate exposures in this study population (Blount et al. 2006a; Steinmaus et al. 2007). These observations suggest one of the following: unexpectedly small increments in IUI may cause thyroid effects, perchlorate may act through additional mechanisms, or the model estimates of IUI are inaccurate.

Although other epidemiological studies with direct measures of exposure have not demonstrated perchlorate-associated thyroid effects, they suffer from a number of limitations compared with the 2001–2002 NHANES analyses. In one case, the cohort included only 16 individuals (Gibbs and van Landingham 2008); two involved cohorts with higher iodine intakes (Amitai et al. 2007; Tellez et al. 2005); and one report lacked sufficient detail to evaluate the analysis (Pearce et al. 2007a). Thus, although the epidemiology is not consistent, studies on the largest cohort did find an association between perchlorate and thyroid effects in women with urinary iodide levels < 100 μg/L at exposures commonly encountered in the U.S. population and well below the NRC RfD. Thus, based on the available data, MA DEP believes that its decision to treat the lowest dose of the Greer et al. (2002) study as a minimal adverse effect is appropriate.

The selection of UFs is open to differing interpretations of the science. The NRC used a UF of 10 to account for human variability and included no other UFs, resulting in an RfD of 0.7 μg/kg/day. The NRC committee itself was not unanimous on this issue, with one member stating that a higher UF (30) was warranted to account for database uncertainties (NRC 2005). This would lead to an RfD of 0.23 μg/kg/day. CalEPA used a UF of 10 for human variability, which yields an RfD of 0.37 μg/kg/day based on their BMDL-derived POD (Ting et al. 2006). The U.S. EPA adopted the NRC RfD. Had the U.S. EPA BMDL estimate been used as the POD, with application of a UF of 10, the RfD would have been 0.20 μg/kg/day. In the derivation of the MA RfD of 0.07 μg/kg/day, a UF of 100 was used to account for uncertainties relating to human variability, minimal LOAEL to NOAEL extrapolation, and database deficiencies.

The differences in the health-based drinking water values between these groups are largely attributable to the use of different RfD and RSC values. Compared with MA DEP, the use of higher RfD values by U.S. EPA and CalEPA allow for greater total exposures and thus higher RSC values. The California drinking water value of 6 μg/L was derived using an RfD of 0.37 μg/kg/day; an RSC of 60%, supported by data from Valentin-Blasini et al. (2005); and the BW and water intake rate of the pregnant woman (Ting et al. 2006). The recent U.S. EPA IDWHA of 15 μg/L for perchlorate was based on the NRC RfD of 0.7 μg/kg/day and an RSC estimate of 60% derived from dietary exposure estimates from the FDA-TDS and NHANES data (U.S. EPA 2008a).

Notably, the document supporting the U.S. EPA IDWHA estimated that perchlorate intake by nursing infants, bottle-fed infants, and children to 2 years of age, attributable to either maternal perchlorate intake and subsequent expression into breast milk, or direct infant consumption of water with perchlorate at 15 μg/L, would substantially exceed the NRC RfD (U.S. EPA 2008a). At the health advisory level of 15 μg/L, a bottle-fed infant would receive a dose of perchlorate five times higher than the NRC RfD, suggesting that a perchlorate advisory level of 3 μg/L would be needed to protect children’s health. However, the U.S. EPA discounted this information, basing their conclusion on the results of PBPK modeling, which was used to predict perchlorate IUI across various life stages (U.S. EPA 2008a). The U.S. EPA noted that the model did not take into account within-group variability in pharmacokinetics, uncertainty in model parameters and predictions, differences in adaptive responses, or responses in those with insufficient iodine intakes. Despite these limitations, the U.S. EPA used the model to predict IUI of 1.1% in the fetus, 1.3% in the 7-day-old nursing infant, and 2.2% in the 7-day-old bottle-fed infant for combined perchlorate intakes from food and drinking water at 15 μg/L (U.S. EPA 2008a). Premature infants, identified as a particularly vulnerable group (NRC 2005), were not addressed. The U.S. EPA concluded that these IUI levels were not significant. However, these predictions provide little assurance that the IDWHA is health protective for the iodide-deficient fetus and neonate because the model uncertainties are substantial and because animal data (U.S. EPA 2002c) and human data (Blount et al. 2006a, 2007) suggest that perchlorate may affect thyroid function at doses predicted to cause little IUI (U.S. EPA 2008a). Thus, based on current information, the IDWHA may not sufficiently protect sensitive life stages.

More recently, the U.S. EPA Office of the Inspector General (OIG) completed a draft assessment that used a cumulative risk approach to derive estimated NIS inhibitor loads attributable to exposures to perchlorate, nitrate, and thiocyanate (U.S. EPA 2008b). Although aspects of the OIG’s assessment have merit, external scientific review has not yet been completed. Briefly, the OIG estimated that perchlorate exposure at the NRC RfD would contribute < 1% of an adult’s typical total NIS inhibitor load. The OIG draft report also evaluated studies on nitrate- and thiocyanate-exposed populations and concluded that they, on a perchlorate-equivalent exposure basis, support the NRC perchlorate RfD. However, the limitations of the epidemiological studies included were not fully considered, and the results of the Blount et al. (2006a) and Steinmaus et al. (2007) analyses were not addressed.

If the OIG report’s analysis of NIS inhibitor intake is correct, exposures to thyroid toxicants may, based on NIS inhibition, be too high for a significant number of people. As a public health response, the OIG concluded that

the most effective and efficient approach for reducing health risks of permanent mental deficits in children from low maternal thyroid iodide uptake during pregnancy and nursing (for example, attributable to exposures to perchlorate, other NIS inhibitors, and iodide insufficiency) is to add iodide to all prenatal vitamins.

We believe that this is an insufficient response because, in the case of contaminated water supplies, it shifts the responsibility for protecting public health away from those responsible for environmental pollutants to the individuals who bear the risk. Additionally, under this intervention approach, protection of infants from adverse health effects attributable to perchlorate-contaminated water supplies would be completely dependent on the mother’s ability to achieve the necessary iodide supplementation. We believe that more comprehensive policies to reduce exposures to thyroid toxicants are also needed.

## Conclusions

MA DEP used a weight-of-the-evidence approach using mode-of-action, human, and animal data to assess perchlorate risk and selected data from the Greer at al. (2002) study to derive an RfD. Because of potential neurodevelopment risks to children, scientific uncertainties were addressed in a health-protective manner. MA DEP identified the lowest dose level in the Greer et al. (2002) study as a minimum LOAEL, which was used as the POD to derive an RfD. A number of limitations and uncertainties in the perchlorate database were identified and a range of UFs were considered to address these. The MA DEP SAC supported UF values ranging from 30 to 300, with associated RfDs of 0.23 μg/kg/day and 0.02 μg/kg/day. MA DEP scientists and most SAC members concluded that a composite UF of 100 was most appropriate, resulting in a final RfD value of 0.07 μg/kg/day. Data from animal studies support a similar or perhaps even lower value [see Supplemental Materials, Section C (doi:10.1289/ehp.0900635.S1)].

To account for other sources of exposure, MA DEP applied a default RSC of 20% to derive a health-based drinking water value of 0.49 μg/L for adults. More recent data indicate that food exposures to perchlorate are close to, or even exceed, the MA RfD (U.S. EPA 2008a), which would support a lower RSC. The final MA DWS for perchlorate was set at 2 μg/L based on a risk management decision to minimize potential compliance issues at PDWS attributable to perchlorate in chlorination disinfection treatments. A standard of 2 μg/L minimizes possible disincentives to adequate chlorination, which could raise drinking water risks from pathogens. MA DEP concluded that this standard reasonably balanced potential perchlorate exposure and infectious disease concerns and was also within the range of scientific uncertainty regarding perchlorate toxicity.

Estimates of exposures to perchlorate and other thyroid toxicants, combined with data indicating that significant numbers of U.S. women are iodide insufficient, point to the need for more comprehensive approaches and policies to address and reduce exposures to thyroid toxicants in drinking water, breast milk, and food and to improve iodide intake. Pending further scientific developments, including explanation or confirmation of the observations of Blount et al. (2006a) and Steinmaus et al. (2007), we believe that the MA RfD and DWS are supported by the available data and, compared with current U.S. EPA values, better protect children’s health.

## Figures and Tables

**Table 1:** Perchlorate concentrations in sodium hypochlorite (NaOCl) solutions.

Source	No. of samples	Perchlorate (μg/L)		Comments
		Mean	Range	
Drinking water treatment plant newly delivered 15% NaOCl solution	1	< 0.2	Not applicable	
Drinking water treatment plant 26-day postdelivery 15% NaOCl	5	2,461	490–6,750^a^	
Household bleach	5	1,834	89–8,000^a^	Highest values from oldest bottle
Wastewater treatment plant	3	2,753	260–4,600^a^	
15% NaOCl	3	2,163	900–4,100^b^	

a Analyzed using U.S. EPA method 331.0 (U.S. EPA 2005b).

b Analyzed using U.S. EPA method 314.0 (U.S. EPA 1999).

**Table 2:** Perchlorate RfDs and drinking water values by various agencies.

Parameter	MA DEP	NRC majority	U.S. EPA	CalEPA
POD	Greer et al. (2002) low-dose group as minimum LOAEL	Greer et al. (2002) low-dose group as no effect level	Adopted NRC value	Greer et al. (2002) BMDL at 5% IUI
UF	100	10 (30^a^)	Adopted NRC value	10
Basis of UF	Minimum LOAEL to NOAEL; sensitive subgroups; data gaps	Sensitive subgroups	Adopted NRC value	Sensitive subgroups
RfD (μg/kg/day)	0.07	0.7 (0.23^b^)	Adopted NRC value	0.37
Relative source contribution factor	20%	Not calculated	60%	60%
Adult drinking water value (μg/L)	0.49 (2^c^)	Not calculated	15	6^d^
Infant drinking water value (μg/L)	0.43^e^	Not calculated (4.3^e^)	Not calculated (3^f^)	Not calculated

a One member of the NRC and SAC supported a UF of 30 (MA DEP 2006; NRC 2005).

b RfD using UF = 30.

c MA DWS was based on risk management considerations.

d The California Public Health Goal was based on an RSC of 60% and BW and water consumption rate of the pregnant woman (Ting et al. 2006).

e Drinking water value calculated by MA DEP using infant BW of 4 kg, water consumption of 0.64 L/day, and RSC = 100%.

f Drinking water value necessary for bottle-fed infants to meet NRC RfD based on exposure estimatea in U.S. EPA (2008a).
